# Visual recognition for urban traffic data retrieval and analysis in major events using convolutional neural networks

**DOI:** 10.1007/s43762-021-00031-w

**Published:** 2022-01-06

**Authors:** Yalong Pi, Nick Duffield, Amir H. Behzadan, Tim Lomax

**Affiliations:** 1 Institute of Data Science, Division of Research, Texas A&M University, College Station, TX 77843 USA; 2 Department of Electrical and Computer Engineering & Institute of Data Science, Texas A&M University, College Station, TX 77843 USA; 3 Department of Construction Science, Texas A&M University, College Station, TX 77843 USA; 4 Texas A&M Transportation Institute, Texas A&M University System, College Station, TX 77843 USA

**Keywords:** Computer vision, Object detection and tracking, Monitoring visual data, Traffic volume, Game-day traffic, Intersection turning

## Abstract

Accurate and prompt traffic data are necessary for the successful management of major events. Computer vision techniques, such as convolutional neural network (CNN) applied on video monitoring data, can provide a cost-efficient and timely alternative to traditional data collection and analysis methods. This paper presents a framework designed to take videos as input and output traffic volume counts and intersection turning patterns. This framework comprises a CNN model and an object tracking algorithm to detect and track vehicles in the camera’s pixel view first. Homographic projection then maps vehicle spatial-temporal information (including unique ID, location, and timestamp) onto an orthogonal real-scale map, from which the traffic counts and turns are computed. Several video data are manually labeled and compared with the framework output. The following results show a robust traffic volume count accuracy up to 96.91%. Moreover, this work investigates the performance influencing factors including lighting condition (over a 24-h-period), pixel size, and camera angle. Based on the analysis, it is suggested to place cameras such that detection pixel size is above 2343 and the view angle is below 22°, for more accurate counts. Next, previous and current traffic reports after Texas A&M home football games are compared with the framework output. Results suggest that the proposed framework is able to reproduce traffic volume change trends for different traffic directions. Lastly, this work also contributes a new intersection turning pattern, i.e., counts for each ingress-egress edge pair, with its optimization technique which result in an accuracy between 43% and 72%.

## Introduction

Major events such as concerts, sports, conferences, and festivals have traffic concentrated in both time and space (Chen & Zhou, 2010), challenging transportation planners and event organizers to mitigate the congestion and ensure safety. Accurate and prompt traffic data, especially in intelligent transportation systems (ITSs), is one of the foundations of successful traffic management (Lin et al., 2017). The main traffic data statistics include speed (distance per unit time), volume (vehicles per unit time), density (vehicle per unit distance), occupancy (percent of time of a point occupied by vehicles), and others. Besides direct traffic information for monitoring, these measurements support traffic modeling so that the hypothetical simulations and predictions can inform the traffic design, management, and decision making. The measurement location could be from a point, a short section (within 10 m), or a long distance (more than 0.5 km). Besides human count (Toth et al., 2013), the existing data collection methods use a wide range of techniques (Leduc, 2008). Sensor based methods such as induction loops, pressure hoses, electric sensors, and radars are designed with particular equipment to detect passing vehicles. The main drawback of these approaches is they require high-cost installation and maintenance (Yang & Pun-Cheng, 2018). Cellular GPS data are another emerging source of traffic trip data. However, it is challenging to label the transport modes, e.g., differentiate a driver data point from a passenger (Sadeghian et al., 2021). Thus many researchers have used the public available taxi GPS data to sample the total traffic scenario. Computer vision using the existing cameras and computers is another popular method due the low cost. Both traditional and artificial intelligence (AI) based computer vision identify visual signals from the camera and perform vehicle counting, incident detection, and speed measurement. However, the challenges remain in the visual input aspect, e.g., low resolution cameras and object occlusion, and the environmental aspect, e.g., lighting condition (day and night) and weather (rain, fog, and snow). Altogether, these data collected by various means are processed and analyzed for application such as traffic monitoring and documentation, risk alert system, simulation and modeling, and ITS.

Especially in major events, real-time or near real-time traffic data are critical to safety management and congestion mitigation. With the development of the optical cameras and graphic processing unit (GPU), more and more low-cost computer vision techniques are implemented in domains of traffic management. However, there is still a limited body of work in the design and evaluation of AI computer vision for major events traffic volume count. Besides, the intersection turning pattern remains unexplored, especially the left turning traffic which requires more time to move through an intersection. The detailed understanding of the intersection traffic can assist local management to mitigate congestion by controlling (i.e., opening, closing, and combining) lanes, garage driveways, and roads. Furthermore, traffic monitoring cameras, as the main source of the visual signals, need to function under various input resolutions, lighting conditions, camera angles, and object sizes. Therefore, there is a need of investigation of such influencing factors. The study of these parameters and their recommendations is beneficial to the practitioners when adjusting and installing cameras.

To address these challenges, this paper reports a framework designed to extract granular traffic data from video imaging originated from cameras operated by the Texas A&M University (TAMU) Transportation Services in College Station, Texas. The proposed framework is tested on a regular school day and four TAMU home football games against Vanderbilt, Florida, Arkansas, and Louisiana State University in the 2020 college football season. The regular school day experiment is carried out to optimize the input resolution and confidence threshold (see definition in Section 4) under various lighting conditions (samples from a 24-h-period). Moreover, by dividing the camera view into multiple study areas, parameters including camera angle and pixel size are also optimized. With these optimized parameters, the framework is then tested on the four game-day traffic videos to extract granular time series of vehicle volume counts on University Drive (a major traffic road near Kyle Field, home of TAMU football team). These results are compared with previous game-day reports published by the Texas A&M Transportation Institute (TTI). With the same parameters, this research also proposes an intersection turning pattern matrix that is not available previously, hence one of the contributions of this work. This matrix is comprised of ingress-egress combinations marked by the four intersection edges. Based on edge traffic count, an optimization technique is designed and compared with the ground truth data manually labeled by a trained researcher, under the supervision of a senior TTI researcher. The practice of using humans to count traffic data from the recorded video data can be found in other research (Koita & Suzuki, 2019; Toth et al., 2013). Moreover, it is a common practice in computer vision to use humans to generate the ground truth data and compare it with the machine performance (Buch et al., 2011; LeCun et al., 2015). The final goal is to extract vehicle data from which aggregate traffic statistics can be computed to inform game day traffic management and operations. The primary contributions of this study to both retrospective analysis and real-time traffic management include:

- A traffic data retrieval framework which can produce data including vehicle location, direction, speed, and time.
- Investigation of the influence of factors including lighting condition, pixel size, and camera angle on the counting performance.
- A unique homography decomposition method to estimate the camera angles based on four reference points.
- Detailed metric, analysis, and optimization for intersection turning pattern detection based on the traffic volume count.

The rest of this article is organized as follows: Section 2 contains the literature review of the related work, before Section 3 describes the methodology and workflow of the proposed framework. Section 4 explains the traffic volume count experiment, performance calculation, and the influencing factor analysis. Section 5 presents the traffic volume analysis after four football games compared with TTI reports (seasons 2014–2020). Section 6 contains the design, measurement, and optimization of the intersection turning pattern. Lastly, Section 7 provides the conclusion and a discussion on potential directions for future research.

## Literature review

In the domain of traffic management, some studies have explored techniques to collect and analyze the traffic data. One recent research has studied the traffic data (collected by in-road detectors) around the Los Angeles Memorial Coliseum during games played by the Los Angeles Rams and the University of Southern California (USC) Trojans (Giuliano & Lu, 2021). The study revealed the relationships between game attributes (attendee, time, and distance) and traffic performance. Based on the results, two strategies to smooth the traffic are suggested: rewarding Rams attendees to arrive early and extending traffic control to nearby freeway interchanges. TTI had been conducting game day traffic data collection and analysis around College Station since 2014 (Texas A&M Transportation Institute, 2014, 2015, 2016, 2017, 2018, 2019, 2020). In these reports, the congestion percentage data are purchased from the commercial company INRIX (Mikulski, 2016) and associated with local traffic management plans. Traffic data providers such as INRIX collect data from multiple main road sensors and vehicles (INRIX, 2021). In a different study, social media data were used to extract traffic data. Natural language processing (NLP) techniques were used to exclude posts that were irrelevant to the events (Ni et al., 2014). The remaining location data of the users were then used to estimate the traffic using neural network. Another research group examined a method that uses real-time spatial-temporal data to forecast traffic 5 min into the future. The following results showed a 80% accuracy compared with data collected by loop detectors (Min & Wynter, 2011). Altogether, there are various data collection methods for both major events and regular traffic. Some of them rely on physical sensors or equipment and the others rely on noisy user-generated data (social media).

Different from the abovementioned approaches, advances in computer vision has led to less resource-demanding alternatives using the existing computers and cameras (Leduc, 2008). Convolutional neural network (CNN) is a type of feed forward network (10–20 layers) that is designed to process multiple arrays (e.g., colored images). Especially in the computer vision domain, the convolutional filters inside CNNs are the keys to extract edge, color, and pattern (LeCun et al., 2015). In tasks such as classification (Lu & Weng, 2007), object detection (Zhao et al., 2019) and semantic segmentation (Garcia-Garcia et al., 2018) CNNs tend to outperform traditional techniques with various camera parameters such as focal length and resolution. Moreover, CNN models can cover multiple classes simultaneously such as car, pedestrian, and bus as long as they appear in the training dataset (Everingham et al., 2015).

In a recent study, a CNN traffic classification model, which detects traffic density from the input image, was proposed and achieved 99% overall accuracy (Zhou et al., 2017). Another work reported 80% accuracy in traffic congestion classification using CNN models on non-laned traffic images (Chauhan et al., 2019). Different from image classification, object detection is capable of extracting more details (bounding boxes) from the camera. One study employed fast region-based CNN (fast-RCNN) and counted cars individually with a 96% precision in 100 traffic images (Zhang et al., 2016). Another example integrated deep neural network with long-short-term-memory (LSTM) networks (Zhang et al., 2017a) and achieved absolute errors (missing vehicle per image) of 4.21 and 1.53 on public available datasets TRANCOS (Onoro-Rubio & López-Sastre, 2016) and WebCamT (Zhang et al., 2017b), respectively. Some researchers had combined segmentation CNN model (Mask-RCNN) and object tracking algorithm to count vehicles and accomplished 98% precision (Al-Ariny et al., 2020). A different study had focused on anomaly detection such as stalling in traffic flows. This work used CNN and background subtraction and achieved high accuracy (Shine et al., 2019). A recent research combined CNN, LSTM, and transpose CNN to predict traffic congestion based on a series of abstract map images and showed 86% accuracy on a four-hour-long video in Seoul (Ranjan et al., 2020). Altogether, throughout the literature review, there is a little amount of work focusing on major event traffic volume count and intersection turning pattern detection using CNN computer vision techniques.

## Methodology

**Fig. 1:**
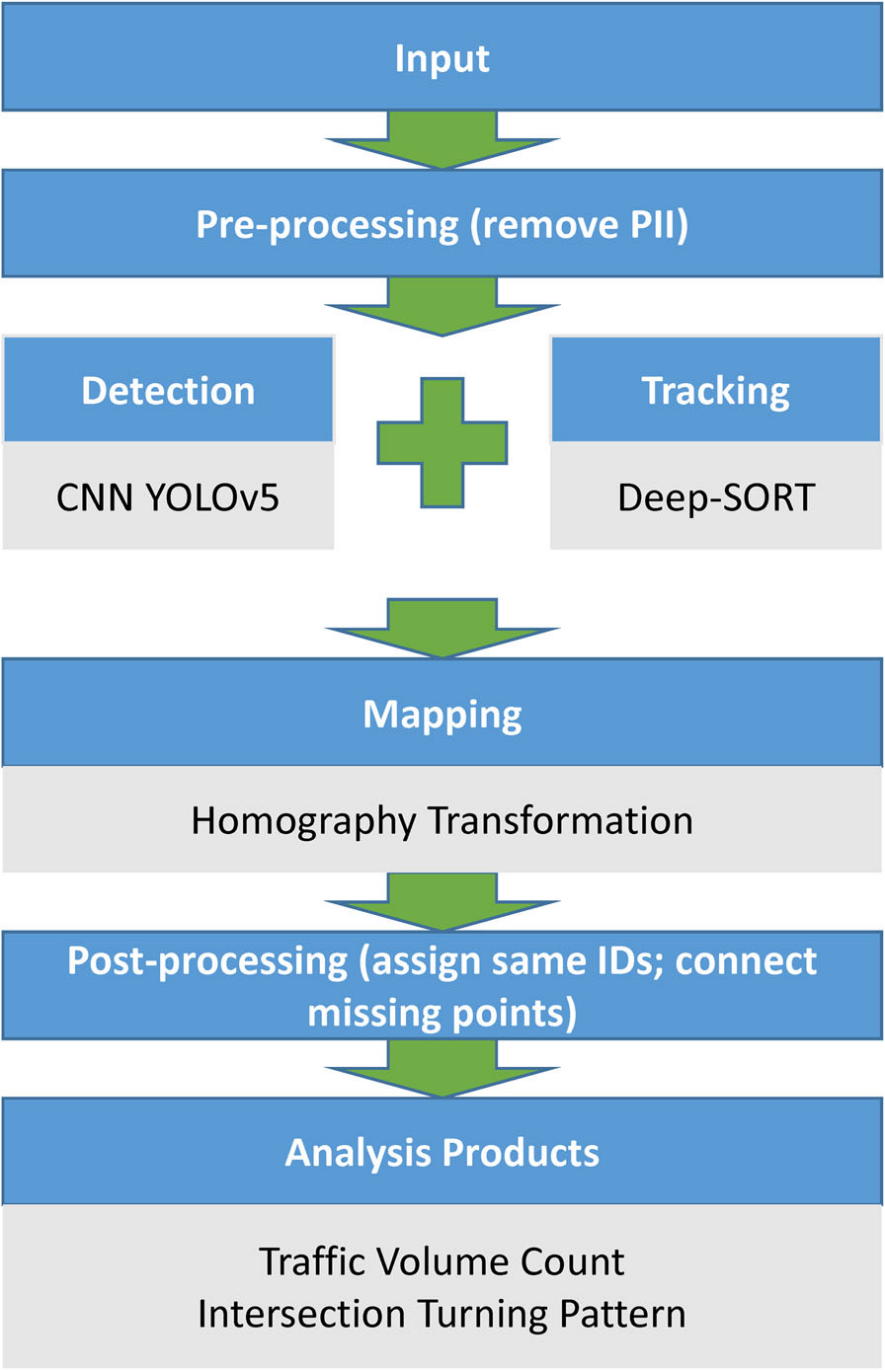
System workflow

The workflow of the proposed framework is displayed in Fig. 1. First, the framework takes a monitoring video as input, pre-processes this input to remove personally identifiable information (PII) such as license plate numbers and human faces. Next, each frame of the video is passed on to a CNN model, namely YOLOv5 (you-only-look-once version 5) to detect the vehicles. YOLOv5 (Jocher, 2020) is a CNN model that takes a digital image and outputs bounding boxes that surround the objects. In this work, the training dataset for YOLOv5 is the common objects in context (COCO) dataset, which is an annotated image dataset containing 328,000 images with 80 common classes, e.g., car (Lin et al., 2014). The reasons for choosing YOLOv5 come in two folds. The first is that YOLOv5 is one of its latest versions that is capable of achieving a mean average precision (mAP) of 69.6% on COCO testing dataset. This result is better than the previous YOLO versions (Redmon & Farhadi, 2018, 2016) and others competing CNN models (He et al., 2017; Ren et al., 2015). The second is that YOLOv5 is capable of processing image in real-time or near real-time speed depending on the hardware. The end-to-end processing speed in this work is tested on a consumer grade computer with a Intel i9 central processing unit (CPU), 32 GB memory, and a NVIDIA Quadro T2000 GPU. The results show an average processing speed of 2.09–3.6 frame per section (FPS). However a higher performance GPU or multi-GPU framework will facilitate the speed up to real-time speed (15 FPS monitoring video in this work).

However, the detected bounding boxes are in individual video frames (images), i.e., they are not connected hence cannot capture vehicle traces. To remedy this problem, the output of YOLOv5 is further integrated with an object tracking algorithm Deep-SORT (Bewley et al., 2016). Deep-SORT works side-by-side with an object detection CNN model (YOLOv5 in this study) to track objects in sequential frames. There are three main components in Deep-SORT: Hungarian assignment algorithm to assign unique IDs to each object, CNN feature extractor to re-identify the same object, and Kalman filter to smooth the traces. This study uses the standardized multi-object tracking accuracy (MOTA) established by the multiple object tracking (MOT) challenge (Milan et al., 2016) to measure the tracking performance. MOTA measures the true positive tracks and IDs over the total ground truth, and is one of the most widely used metrics in the computer vision tracking research community. Deep-SORT can achieve a high 61.4% MOTA on MOT challenge with a processing speed at the rate of 260 Hz. Hence it is chosen to be integrated with YOLOv5 in this work. However, the original Deep-SORT CNN feature extractor was trained on a mixed dataset of pedestrian (majority), car, bike, and motorcycles. For better results, the Deep-SORT CNN feature extractor in this work is re-trained on the VeRi dataset (Liu et al., 2016) resulting in a MOTA of 60.54% when tested on our own dataset. The error margin is set to 3.05 m assuming 10-ft wide traffic lanes, which is equivalent to ±1 traffic lane. VeRi dataset is a vehicle re-identification dataset that contains 50,000 images of 776 vehicles captured by 20 cameras in 24 h. It is worth to note, the original 61.4% MOTA of Deep-SORT was based on 100% accurate detection provided by the challenge organizers (Leal-Taixé et al., 2015). In comparison, the MOTA in this study is based on the YOLOv5 detections which already have 25–30% false positive cases. Considering this, the proposed framework is able to successfully track vehicles with high accuracy.

**Fig. 2:**
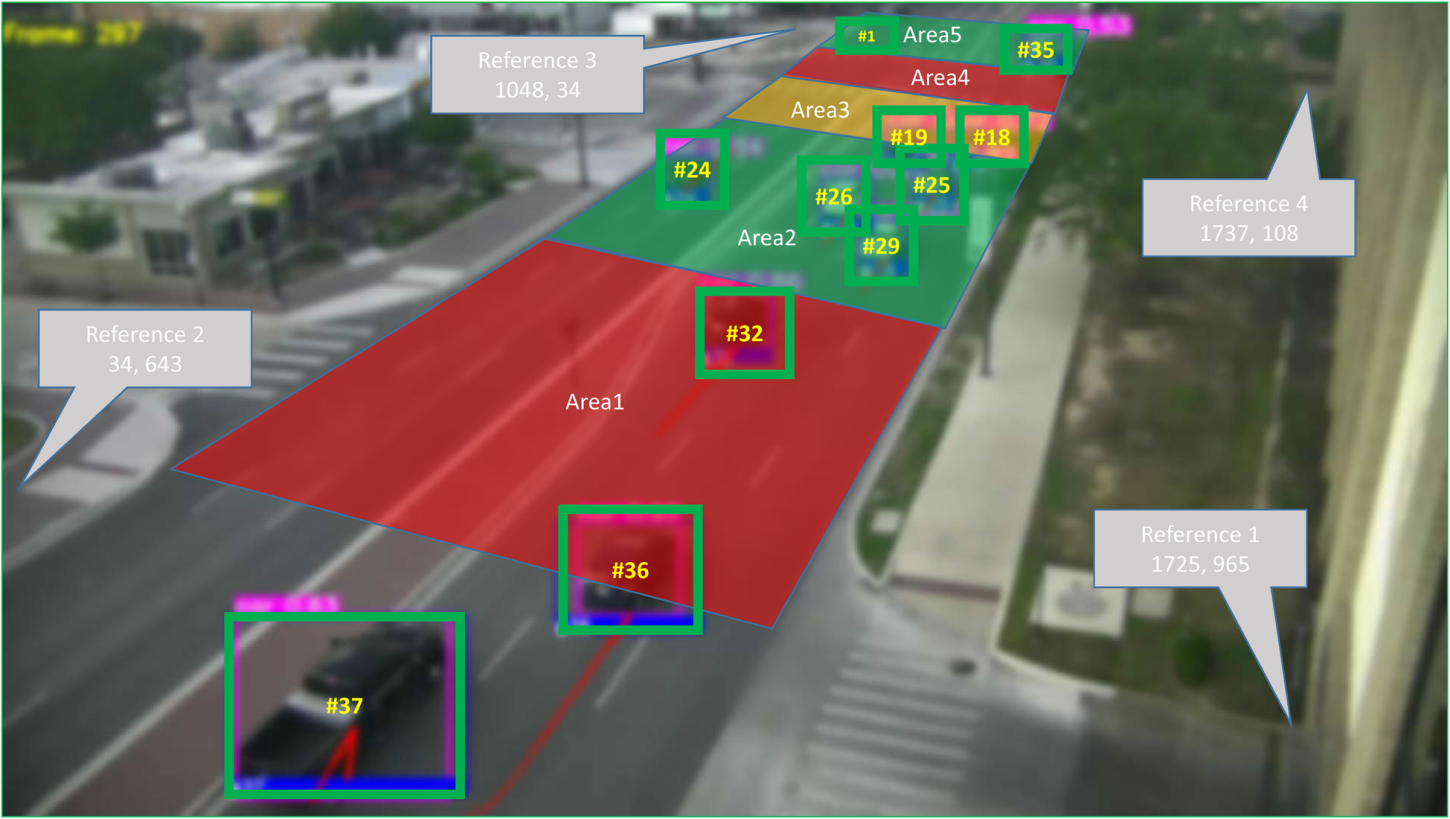
Reference points and Areas 1 through 5 in the camera view. Each bounding box indicates a detected vehicle with assigned ID # (image is blurred for privacy preservation)

Figure 2 shows the output bounding boxes with unique IDs for each observed vehicle. In this example frame, each box indicates a detection with the assigned ID #. Besides the bounding box information, each image is blurred (pre-processing) as shown to remove the PII, which is a non-reversible process, i.e., the underlying image cannot be reconstructed. The bottom center of each bounding box represents the pixel location of a vehicle in the camera. However, it does not support further analysis such as speed calculation or traffic direction sensing in a real-world scale. Consequently, coordinate mapping using homography transformation is carried out to project the assigned IDs and pixel locations onto an orthogonal map. In this research, the map system is the Universal Transverse Mercator (UTM) system in meters, which is a common practice in maps such as Google Maps. The UTM zone code of College Station city is 14R. Equation 1 expresses the homographic function used to project a point from the input plane onto the output plane. Here, the input plane is the camera view and the output plane is the orthogonal UTM map. In Eq. 1, *H* is a 3*3 transformation matrix written in Eq. 2. (*X*_*input*_, *Y*_*input*_) and (*X*_*output*_, *Y*_*output*_) are the coordinates of the same point on each plane. While projecting without scale effect, *i* in Eq. 2 is equal to 1, leaving 8 variables to solve in *H*.

1 $$ \left|\begin{array}{c}{X}_{output}\\ {}{Y}_{output}\\ {}1\end{array}\right|=H\ast \left|\begin{array}{c}{X}_{input}\\ {}{Y}_{input}\\ {}1\end{array}\right| $$

2 $$ H=\left|\begin{array}{ccc}a& b& c\\ {}d& e& f\\ {}g& h& i\end{array}\right| $$

Four pairs of reference points in both planes, displayed in Figs. 2 and 4 as reference 1–4, are manually selected and measured to solve *H*. In this work, the reference points are selected by the authors who are familiar with the site and have done similar works (Pi et al., 2020b; Pi, Duffield, et al., 2021; Pi, Nath, et al., 2021). The selection is recommended to be easy-to-identify points such as a pavement corner, the end of a traffic light pole, or the bottom of a building corner. The generalizable rule for selecting the reference points is that the four reference points should be coplanar since homography transformation projects from one plane to another (Pi, Nath, et al., 2021). With a solved *H*, any newly detected vehicle, e.g., bounding boxes in Fig. 2, can be projected onto the map using Eq. 1. It is important to note, *H* only needs to be computed once as long as the camera is stationary, i.e., a new camera position and/or angle requires a unique set of four reference points. The shaded study areas Area 1–5 in Fig. 2, which are manually defined areas for the performance analysis (Section 4), are also projected onto the UTM map as shown in Fig. 4. Meanwhile, the time stamp of each frame is obtained using the video FPS rate and starting time. Altogether, the projected UTM map information includes the ID, location, and time for each vehicle in each frame.

The post-processing of the framework output contains two steps. The first one is to assign the same ID to the circles in two neighboring frames when they have a distance smaller than 1.84 m (average distance measured from the ground truth). The second one interpolates the missing circles that share the same ID. It is worth noting that connecting missing circles of a blocked vehicle (e.g., occluded by trees or traffic lights) reflects the true trajectory making the analysis more useful, but lowers the MOTA measurement. The reason is that MOTA is computed as the percentage of true track points divided by the ground truth points (i.e., tracks that are observed and manually labeled). While generating the ground truth, the occluded vehicles are not labeled hence decrease the MOTA measurement.

**Fig. 3:**
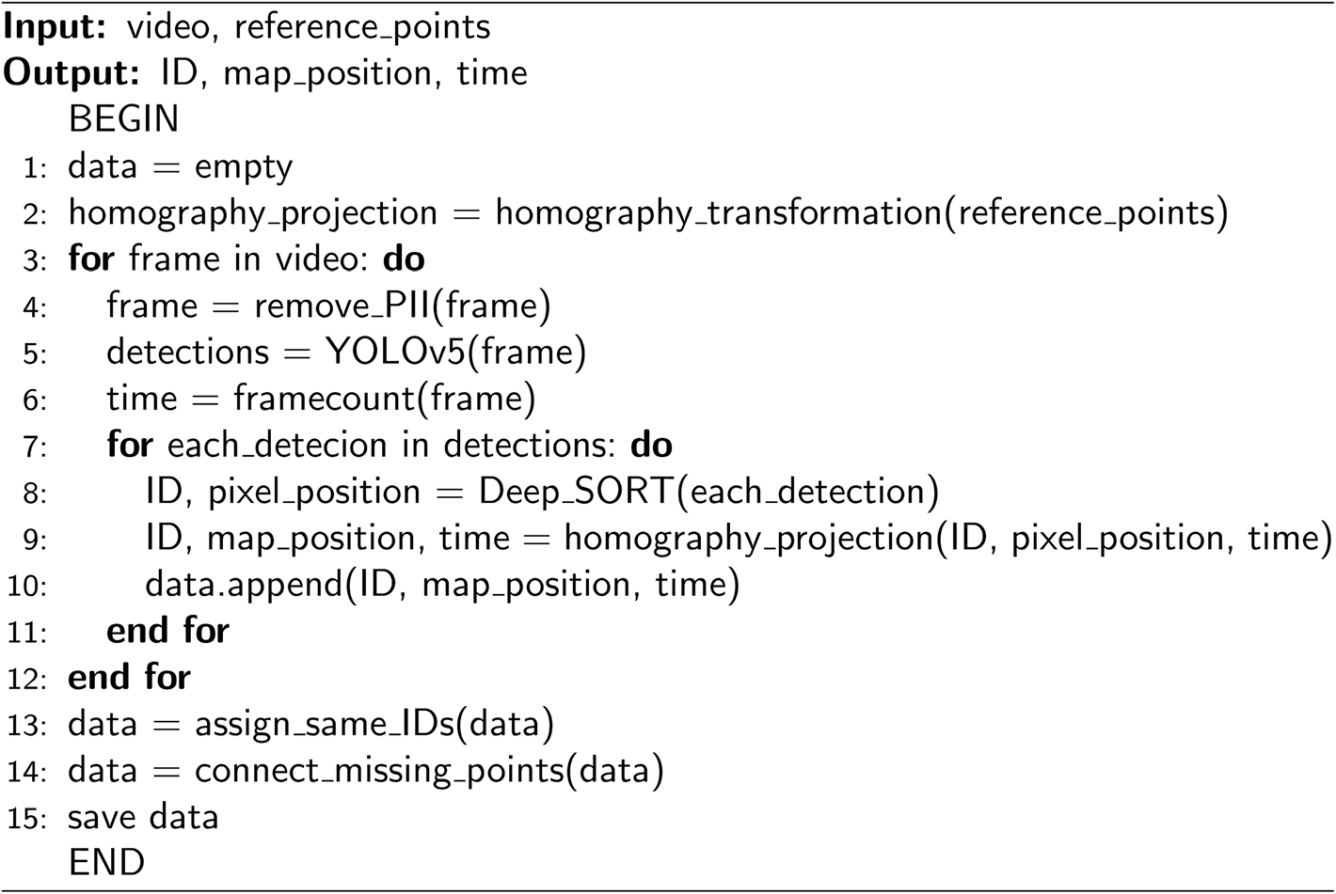
Framework pseudo code

To further display the structure of this framework, Fig. 3 illustrates the pseudo code of the workflow. The main requirement for this framework is open source programming tool such as Python and consumer or above grade computers. In this framework, the input data are the *video* and the manually selected four pairs of *reference points*. The reference points only need to be defined once to work for other videos recorded from the same camera position. Each frame of the video is processed by function *remove_PII* to anonymize first, before function *YOLOv5* processes it and outputs the detections. Next, function *Deep_SORT* tracks data including *ID, pixel_position,* and *time*. Functions *homography_projection* is established by taking four *reference_points* (line 2), and it is being applied on the output of *Deep_SORT*. The projected data are then appended and saved for post-processing functions *assign_same_IDs* and *connect_missing_points*. After post-processing, this data are ready for further domain specific analysis such as traffic management.

**Fig. 4:**
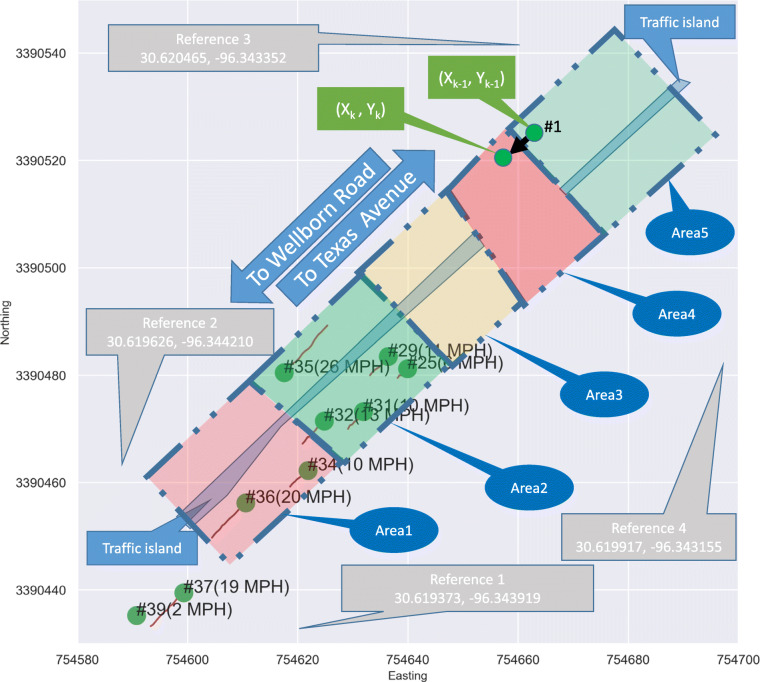
Reference points, Areas 1 through 5, and traffic projection on UTM map. Each circle represents the projected vehicle. The line connected to the circle indicates its trace in the last second, which is used to compute the mile per hour (MPH) next to the ID #

Figure 4 visualizes every vehicle (circle) with a unique ID and a line representing its location in the last second. Based on this, the speed (miles per hours, a.k.a., MPH) is computed and marked in the parentheses next to the ID. Furthermore, the direction of each vehicle, i.e., to Texas Avenue or to Wellborn Road, can be determined by this trace. For the analysis products, a counting mechanism is established to count the event when an object traverses a pre-defined area as follow. Let trace *T* = [(*X*_*k* − 1_, *Y*_*k* − 1_), (*X*_*k*_, *Y*_*k*_)] be its Easting (X-axis) and Northing (Y-axis) coordinates in frames *k* − 1 and *k*. For each defined area *a*, the count *C*^*a*^ increases by 1 unit if *T* crosses any edge of *a*. If the point (*X*_*k*_, *Y*_*k*_) is located inside of *a*, *C*^*a*^ is defined as ${C}_{ingress}^a$. On the other hand, when the point (*X*_*k*_, *Y*_*k*_) is located outside of *a*, *C*^*a*^ is defined as ${C}_{egress}^a$. If a vehicle does not cross any edge, there is no count change. By iterating through all video frames, the count *C*^*a*^ for area *a* is calculated using Eq. 3. Take vehicle ID #1 in Fig. 4 as an example, it is entering Area 4 from Area 5, thus increasing both ${C}_{ingress}^4$ and ${C}_{egress}^5$ by one unit. In the following Section, this mechanism is used to produce the traffic volume count and intersection turning pattern.

3 $$ {C}^a=\left({C}_{egress}^a+{C}_{ingress}^a\right)/2 $$

## Traffic volume count experiment and analysis

The goal of this experiment is to investigate the performance influencing factors including input resolution, luminance (lighting condition), detection pixel size, and camera angle. Hence, a 24-h-long video data are collected from a camera facing University Drive on May 13, 2020. This day is a regular school day before the football season with clear weather. The camera is the same as illustrated in Figs. 2 and 4, thus the same reference points are used for the homography transformation. By random sampling, 60 1-min-long videos are extracted, and each video frame is manually annotated by drawing bounding boxes with IDs surrounding individual vehicles. Compared with this human-produced ground truth data, the proposed framework is able to detect vehicles with an average precision (AP) of 71.71% and track them with a 60.54% MOTA. Moreover, the best input resolution for class car (vehicle) is measured to be 1280*1280 (Pi, Duffield, et al., 2021), based on the labeled data. Hence, all the following experiments use this input size.

The relative luminance value can be calculated as 0.299 *R* + 0.587*G* + 0.114*B,* where *R*, *G*, and *B* are the respective pixel value in the red, green, and blue channels (Palus, 1998). Of note, this luminance value is not illumination lux; rather, it is a relative measurement to reflect the lighting condition, i.e., bright or dark. Each detection produced by a CNN model comes with a confidence value which describes the probability of the class prediction. In practice, there is a confidence threshold (1–100%) set to exclude low probability detections so the framework performs balanced true and false positive detections (Pi et al., 2020a). To this end, all possible thresholds are tested on the 60 samples and compared with the ground truth to determine the best thresholds. Next, the best thresholds and their sample luminance are clustered using K-means method resulting in two main groups: bright (high luminance) and dark (low luminance) with a Silhouette score of 0.85. In detail, cluster bright has the average luminance of 112.52 with the average threshold of 0.25. On the other hand, cluster dark has the threshold of 0.44 with the average luminance of 79.42. In the following experiments, each frame is clustered using the luminance, followed by selecting the corresponding threshold to filter the low quality detections.

Figure 4 shows five different study areas that are projected and marked with dash lines. The reasons of this study definition (by the same researcher who selects the reference points) are from two aspects. The first one is that these areas are defined along the road to study the effect of the camera angle variance on counting performance. Dividing the areas by the lanes will result in little to none angle variation. The second is that study Area 4 is an intersection that captures traffic not only on University Drive but also two more perpendicular smaller roads. Therefore it needs to be defined individually, hence the rest Area 1, 2, 3, and 5 are defined to cover a similar size space. Moreover, the pixel size of an area *a* is the average size (pixel width*height) of all the bounding boxes (over all samples) that fall inside *a*.

**Fig. 5:**
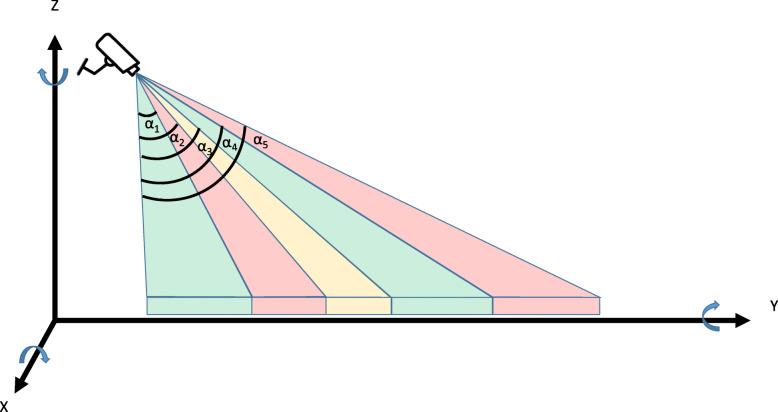
View angle estimation for Areas 1 through 5

Besides pixel size, this research proposes a unique approach to estimate the camera view angle based on homography decomposition. In the pinhole camera model, there is a relationship between real-world 3D coordinates [*X*, *Y*, *Z*] and 2D image coordinates [*x*, *y*], as shown in Eq. 4. Here, *K* is a 3*3 intrinsic parameter matrix containing camera focal length and image center parameters. In Eq. 5, [*R*| *t*] is a 3*4 extrinsic parameter matrix, with *R* being a 3*3 rotation matrix and *t* being a 3-dimensional translation vector. In this experiment, *K* is assumed to be a constant as another study (Malis & Vargas, 2007). Therefore [*R*| *t*] indicates the change from the source coordinate system (3D world) to the destination (2D camera). On the other hand, the 3*3 homography matrix *H*, established in Eqs. 1 and 2, describe the same projection from perspective camera to UTM map. Let [*R*1, *R*2, *R*3] be the three columns of *R* describing rotation as shown in Fig. 5. In this Figure, the arrow on each axis represents the positive rotation direction. Our unique approach assumes both plane-to-plane projections without depth information are the same. Hence there is no *Z* in [*X*, *Y*, *Z*, 1] and no *R*3 in *R*, which leads to *H*^−1^ = [*R*| *t*] in Eq. 6. This gives the values of *R*1 and *R*2 based on the known *H*, thereby *R*3 is the cross product of the two. Next, the complete rotation matrix [*R*1, *R*2, *R*3] is used to derive Euler angles (Slabaugh, 1999), which are the three angles along the X, Y, and Z axis from the source to destination, respectively. For each study area *a*, the corresponding local homography transformation matrix *H*’ is established using the same process. Next, *H*’ is used to estimate the rotation angle from the orthogonal view (facing downward) to the individual camera view marked as *α*_1_, *α*_2_…*α*_5_ corresponding to Area 1 through 5 in Fig. 5. Results show a constant rotation of − 179^°^ on Z-axis and 0.21^°^ on Y-axis for all areas. On X-axis, Areas 1 through 5 have a rotation of 2^°^, 14^°^, 22^°^, 28^°^, and 30^°^, respectively.

4 $$ \left[\begin{array}{c}\mathrm{x}\\ {}y\\ {}1\end{array}\right]=K\left[R|t\right]\left[\begin{array}{c}\mathrm{X}\\ {}Y\\ {}Z\\ {}1\end{array}\right] $$

5 $$ \left[R|t\right]=\left[\begin{array}{cccc}{R}_{11}& {R}_{12}& {R}_{13}& {T}_1\\ {}{R}_{21}& {R}_{22}& {R}_{23}& {T}_2\\ {}{R}_{31}& {R}_{32}& {R}_{33}& {T}_3\end{array}\right] $$

6 $$ {H}^{-1}=\left[R|t\right]=\left[\begin{array}{ccc}{R}_{11}& {R}_{12}& {T}_1\\ {}{R}_{21}& {R}_{22}& {T}_2\\ {}{R}_{31}& {R}_{32}& {T}_3\end{array}\right] $$

**Fig. 6:**
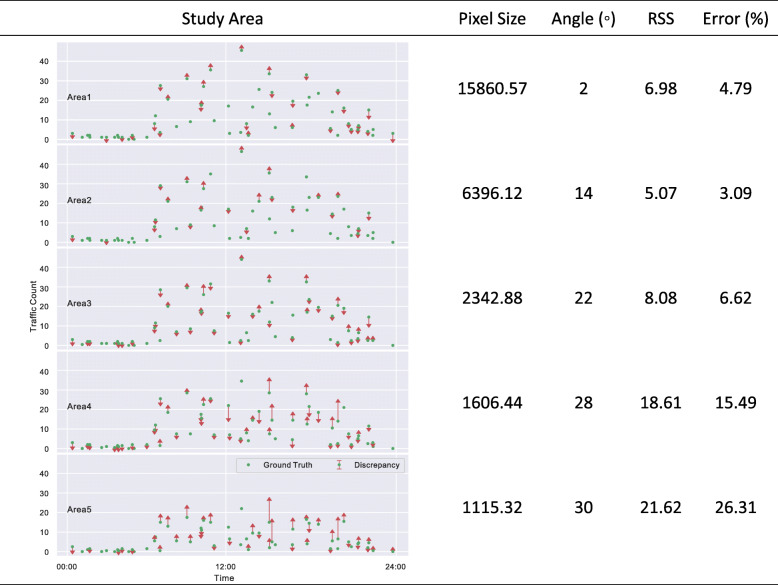
Traffic count performance (RSS and error percentage) and statistics (camera angle and pixel size) for 5 different study areas. In each area subgraph, circles represent the ground truth traffic counts, the corresponding ending points of the arrows indicate the measurements, and the lengths of each arrow are the discrepancies

Let *n* index the 60 samples, hence there are in total *a* ∗ *n* = 5 ∗ 60 = 300 traffic volume count observations. The error *E*^*a*^ for area *a* is computed with Eq. 7, where ${C}_m^{an}$ and ${C}_{gt}^{an}$ are the measurement (*m*) and ground truth (*gt*) count for sample *n*. By definition, this error percentage shows the portion of ground truth that is missed by the framework. Additionally, the root of the sum square (RSS) for each study area is computed using Eq. 8. The RSS indicates the relative discrepancy between the measurement and ground truth. Figure 6 illustrates the experiment statistics including pixel size, view angle, RSS, and error percentage for each area. Moreover, the column “Study Area” has the vertical axis representing the traffic count ${C}_{gt}^{an}$ (circles) and horizontal axis indicating time. In each subgraph, circles without arrows represent cases where ${C}_m^{an}={C}_{gt}^{an}$, i.e., 100% accurate measurement. Upward and downward arrows are overcount (${C}_m^{an}>{C}_{gt}^{an}$) and undercount (${C}_m^{an}<{C}_{gt}^{an}$) cases, respectively, with arrow lengths representing the absolute discrepancy computed as $\mid {C}_m^{an}-{C}_{gt}^{an}\mid$. By definition, the tip of each arrow is the traffic measurement reading of that sample.

7 $$ {E}^a=\frac{\sum \limits_{n=1}^{60} ABS\left({C}_m^{an}-{C}_{gt}^{an}\right)}{\sum_{n=1}^{60}{C}_{gt}^{an}} $$

8 $$ {RSS}^a=\sqrt{\sum_{n=1}^{60}{\left({C}_m^{an}-{C}_{gt}^{an}\right)}^2} $$

According to Fig. 6, Areas 1, 2, and 3 (the areas closest to the camera) have smaller RSS values ranging from 5.07 to 8.08 compared to RSS values of 18.61 and 21.62 for Areas 4 and 5, respectively. Similarly, Areas 1, 2, and 3 bear lower error percentage (3.09–6.62%) compared with Areas 4 and 5 (15.49–26.31%). It is also observed that Areas 4 and 5 have more overcount than undercount, whereas Area 1, 2, and 3 feature a more balanced performance. Moreover, Areas 4 and 5 have larger absolute discrepancy values (longer arrows) compared to Areas 1, 2, and 3, suggesting that Areas 4 and 5 have less stable statistics. Altogether, counting performance has a positive 0.61 Pearson R correlation with pixel size (as objects appear larger, the accuracy improves) and a negative 0.79 Pearson R with view angle (as camera angle increases, the accuracy decreases). On the other hand, the subgraphs show busier traffic between times 6:00 and 22:00 which is consistent with common knowledge. Therefore, it is recommended to define observation areas such that the detection pixel size is above 2343 (rounded pixel size 2342.88 in Fig. 6) and the view angle is below 22^°^. Given the strong correlation, the framework should have a performance similar to Area 1, 2, and 3 with these recommendations.

**Fig. 7:**
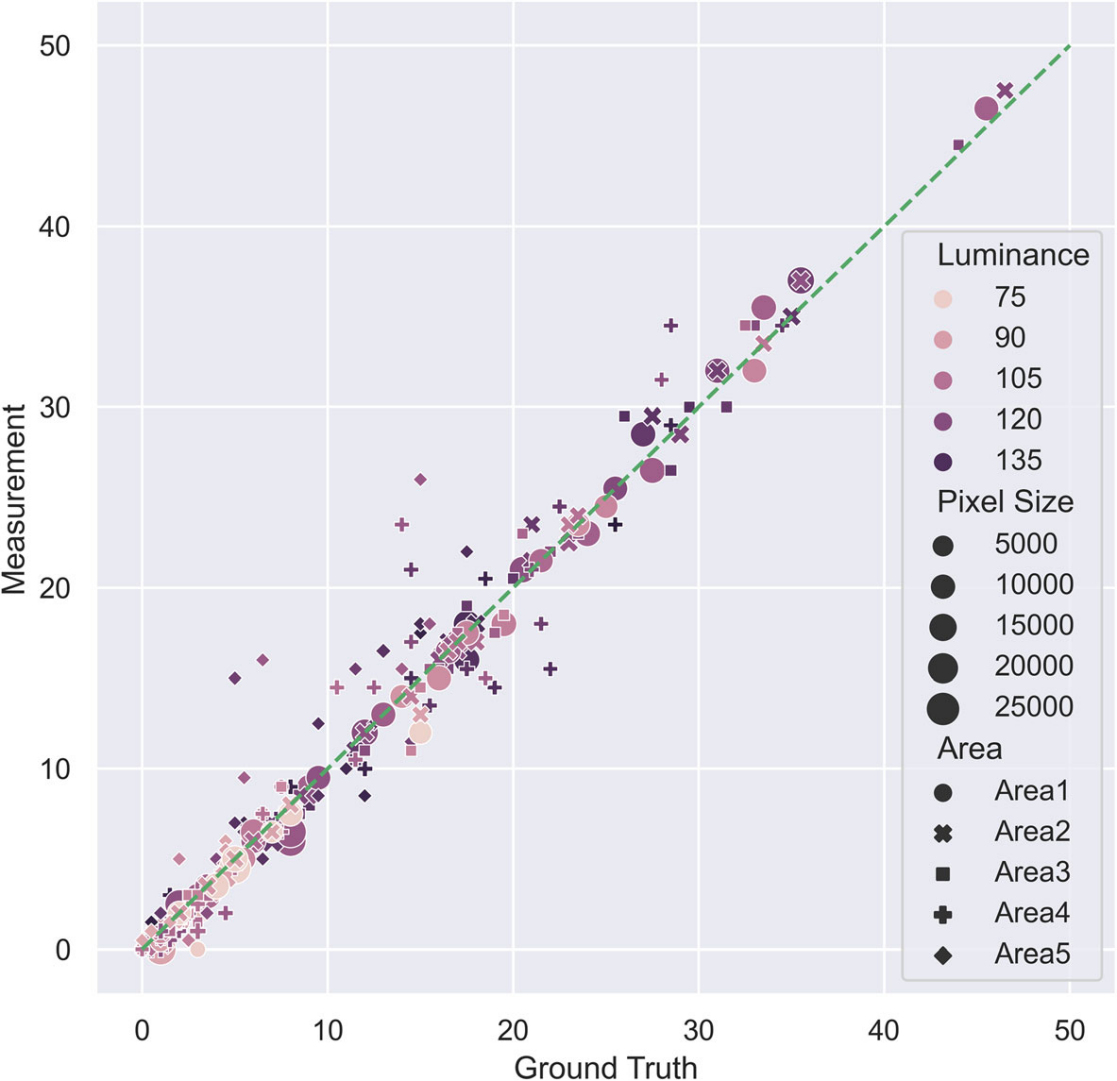
Traffic count precision analysis. Each data point represents the ground truth (X-axis) and measurement (Y-axis) of one sample (60 samples for 5 study areas in total). The icon sizes indicate the pixel sizes. Darker (lighter) icons mean higher (lower) luminance

Figure 7 shows another performance analysis using the same data. In this figure, the horizontal axis is the ground truth count and the vertical axis is the measurement count. Each data point indicates the point of $\left[{C}_m^{an},{C}_{gt}^{an}\right]$. If the framework accuracy is 100%, all 300 points should all lie on the 45-degree equality dash line. Therefore, data points on the upper left and lower right represent overcount and undercount cases, respectively. Each data point is coded with luminance, pixel size and area information. It can be seen that smaller pixel sizes translate into larger overcounts, a pattern that is often observed for Areas 4 and 5. Moreover, higher traffic counts (both ground truth and measurement) are associated with higher luminance readings, i.e., busier traffic during the day. Considering the small divergence in Fig. 7, and the statistics corresponding to the best performing study area (i.e., Area 2 with 5.07 RSS and 3.09% error, as shown in Fig. 6), it can be concluded that the proposed framework is able to count the traffic volume with high precision over a large range of luminance and pixel size.

## Post-game traffic analysis

To test the proposed framework in large events, video data from the same camera during the post-game periods for 4 TAMU football games against Vanderbilt (September 26, 2020), Florida (October 10, 2020), Arkansas (October 31, 2020), and Louisiana State (November 28, 2020) are used to estimate the traffic volume count. These four videos are all the home games (hence have the traffic video recorded) during the 2020 season and they have durations ranging from 1.5 to 2 h. Given the previous study on a 24-h-long data, the best performing Area 2 is selected with the optimized threshold and input size. Next, Area 2 is split into two to sense the directions to Wellborn Road and Texas Avenue with a 5-min interval. This interval selection is to be consistent with the TTI reports for further comparison. Similarly to these reports, the derived traffic data are synchronized considering the ending time of each game, i.e., time 0 is when the game finishes according to the official game information (Texas A&M University, 2020). The videos selected for analysis were only those when the camera was in its “home” location, i.e., no panning or zoom in or out. This feature allows the establishment of one transformation matrix *H* that is applicable to all 4 videos. Nevertheless, the proposed approach can be generalized to moving cameras with more effort to define reference points and study areas for each preset angle.

**Fig. 8:**
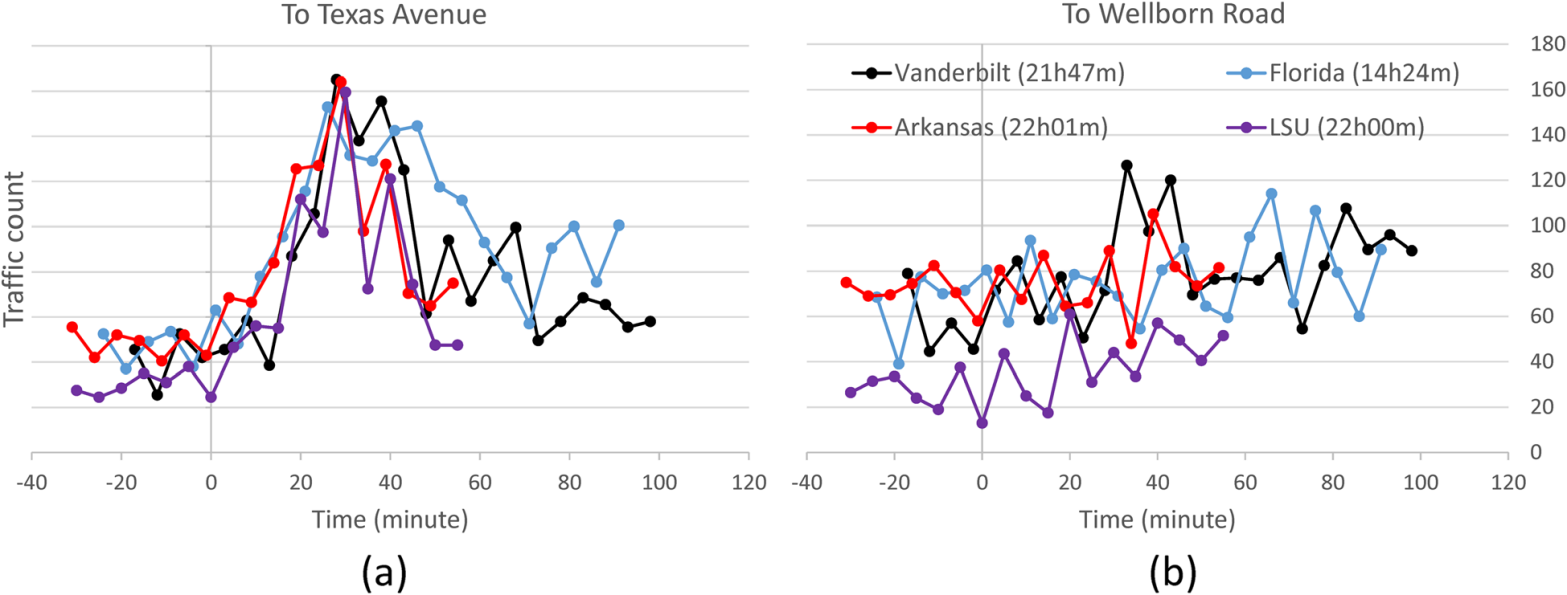
Post-game traffic analysis on University Drive. Legend: opponent team (game ending time). All data are synchronized at the end of the games, denoted by t = 0

Figure 8 shows the traffic volume count on University Drive for eastbound to Texas Avenue in Fig. 8a and westbound toward Wellborn Road in Fig. 8b. In both diagrams, the horizontal axis indicates time (minute) after the end of the game and vertical axis represents the accumulated traffic volume count in 5-min intervals. Comparing all 4 games in both directions, the TAMU vs. Louisiana State game traffic volume is smaller than other games. Possibly due to the absence of other community (non-game-day) traffic during the Thanksgiving holiday. This phenomenon is consistent with the previous TTI reports, where the congestion levels after the Thanksgiving game are the lowest among all games that year.

In the direction to Texas Avenue, the post-game traffic starts to develop immediately after the game and peaks between 20 and 40 min post-game, followed by a diminishing pattern with different rates. This is different from the normal traffic pattern (TTI reports from 2014 to 2019) where the congestion starts at 30 min after a game and lasts for 2 h. The reason behind this is that the seating capacity of Kyle Field was limited to 25% during the COVID-19 pandemic, thus leading to a faster traffic discharge on the adjacent roads. On the other hand, the traffic after the TAMU vs. Florida game, which ends at 14:24 (daytime), diminishes slower than other games that end at night. This traffic pattern is consistent with the TTI report for the 2020 season. According to this report, the reason is that there is more non-game-day traffic after games that end in the afternoon or early evening than games that end in late evenings. In other words, there are more background traffic during the day and early evening, hence the congestion discharge is slower than at night.

In the direction to Wellborn Road, the traffic has no clear peak, which is consistent with previous TTI reports because this direction is generally toward Kyle Field. These non-peak trips are the traffic moving against game-day exit flow, from homes to dining, entertainment, and other destinations (Texas A&M Transportation Institute, 2019). Altogether, it is suggested that the proposed framework can extract the right traffic trend with traffic direction.

## Intersection turning pattern experiment and optimization

**Fig. 9:**
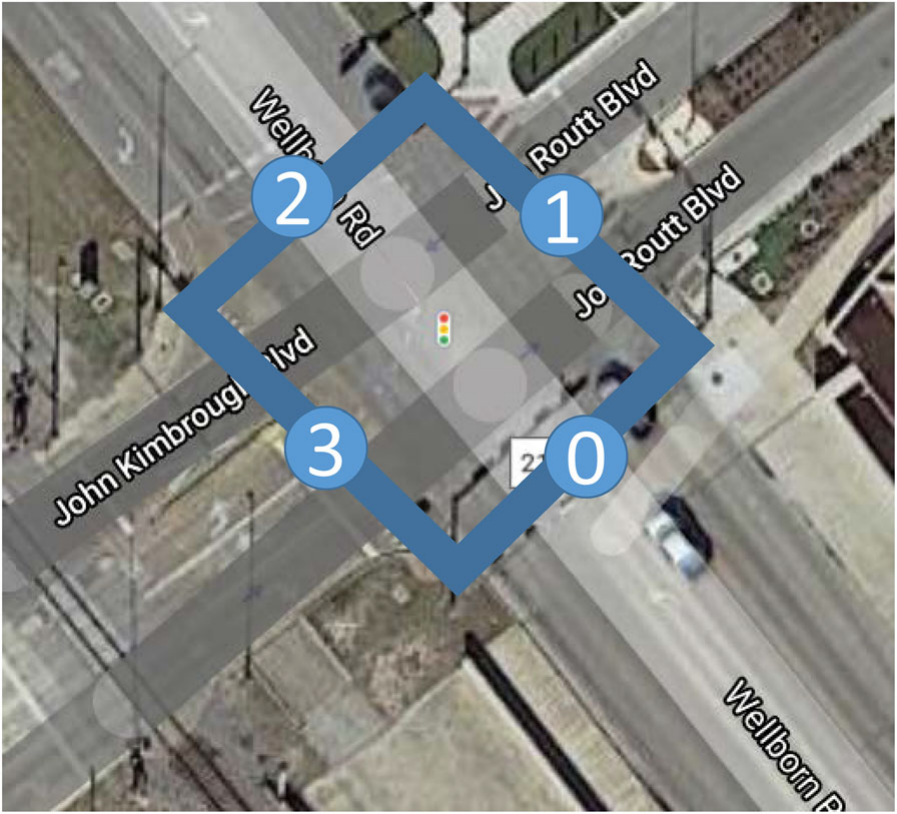
Definition of intersection polygons at the intersection of Wellborn Road and Kimbrough Blvd

The previous experiments showed successful data extraction of traffic volume and trend change on simple straight roads. Another important aspect of traffic management is the intersection, especially major road intersection. A 1-h-long video recorded by a camera on the northwest corner of Kyle Field looking at the intersection of Wellborn Road and Kimbrough Blvd. after the TAMU vs. Florida game is selected to carry out the intersection turning pattern experiment. This camera is different from the previous one, thus necessitating the definition of four new pairs of reference points. The video is divided into 12 samples (numbered #1–12) and annotated by the same researcher as previous experiments under the supervision of a TTI senior researcher. In order to create the turning pattern, the intersection polygon is defined with *d* edges (lines numbered 0 through 3) marked in Fig. 9. Following the same methodology as traffic volume counting (Section 3), events marking when a vehicle enters or leaves the intersection polygon are identified. The origin and destination edge corresponding to each ingress or egress event are recorded in a turning matrix *V* specified in Eq. 9. In this matrix, columns (denoted as *j*) represent the origin edge and rows (denoted as *i*) indicate destination edge. The value of each entry is the traffic count for the paired egress and ingress. For example, *V*_03_ = 10 indicates that 10 vehicles have entered from edge 3 and left at edge 0. Following this process, a ground truth matrix *V*_*gt*_ for each sample is generated using the 12 annotation samples. For each sample, a turning matrix measurement *V*_*m*_ is also produced based on the locations and IDs produced by the proposed framework.

9 $$ V=\left[\begin{array}{cccc}{V}_{00}& {V}_{01}& {V}_{02}& {V}_{03}\\ {}{V}_{10}& {V}_{11}& {V}_{12}& {V}_{13}\\ {}{V}_{20}& {V}_{21}& {V}_{22}& {V}_{23}\\ {}{V}_{30}& {V}_{31}& {V}_{32}& {V}_{33}\end{array}\right] $$

The error percentage between the measurement *V*_*m*_ and ground truth *V*_*gt*_ is calculated similarly to Eq. 7, where *n* is the entry of both matrices*.* However, there is some inconsistency in vehicle ID assignments due to (among others) (i) detection error of dark-colored vehicles, especially on roads with darker surfaces, resulting in vehicle tracks without an origin or destination edge; and (ii) tracking error when the Hungarian algorithm incorrectly assigns the same ID to different vehicles. Considering the precision of vehicle counting earlier, the summation of all the columns and rows of *V*_*m*_ is taken to be sufficiently reliable*.* For each sample, the summation of each column, i.e., the total vehicle amount entering the intersection is defined as *E*. Similarly, the summation of each row, i.e., the total vehicle amount leaving the intersection is defined as *L*. Let *T* = (*E*, *L*) denote the 2*d*-dimensional vector of egress and ingress total counts. Hence, the goal is to produce a *d* ∗ *d* matrix *U* such that the distance between *U* and *V*_*gt*_ is minimized, under the constraint of *T.* Let *V* and *U* concatenate to form *d*^2^ dimensional matrices. Moreover, let *A* denote the 2*d* ∗ *d*^2^ matrix that maps *U* to *T,* i.e., the constraint becomes *AU* = *T.* Hence the problem can be formulated as a scaled least squares norm in Eq. 10,

10 $$ {\left\Vert U-V\right\Vert}_s^2=\sum \limits_{k=1}^{d^2}\frac{{\left({U}_k-{V}_k\right)}^2}{c_k^2}\ \mathrm{s}.\mathrm{t}., AU=T $$

where *k* runs over the paired ingress-egress entries in the traffic matrix. Here, *c*_*k*_ is the “reliability co-efficient” in *C*, which is the *d*^2^-dimensional diagonal matrix with *c*_*k*_, where smaller *c*_*k*_ associates with higher reliability and vice versa. After optimizing each *V*_*m*_, the error of each sample is calculated*.* List of Tables:

Table 1 lists the sample number, post-game time in minutes, summation of *V*_*gt*_, summation of absolute discrepancy, and error percentage. The overcount and undercount are calculated by accumulating the positive and negative elements of *V*_*m*_ − *V*_*gt*_ and listed as well. According to this Table, on average 45% of all turning vehicles are missed by the model. Some samples perform better than others, e.g., sample #2 achieved the lowest error of 28% whereas sample #12 missed 57% of the ground truth. It is also observed that for the same time period, the undercount is more prevalent than overcount (i.e., the framework is missing vehicles more often than logging “phantom” vehicles). Due to tracking error, the accuracy of intersection turning pattern ranges from 43% to 72%.

**Table 1:** Time, ground truth, discrepancy, error, overcount, and undercount for each sample

Sample #	Time (minute)	Sum (*V*_*gt*_)	Absolute discrepancy	Error (%)	Overcount	Undercount
1	5	85	34	40	2	−32
2	10	79	22	28	1	−21
3	15	90	42	47	4	−38
4	20	91	45	49	3	−42
5	25	55	27	49	8	−19
6	30	73	32	44	6	−26
7	35	82	32	39	3	−29
8	40	64	30	47	5	−25
9	45	61	27	44	3	−24
10	50	118	67	57	18	−49
11	55	117	52	44	15	−37
12	60	159	90	57	32	−58

**Fig. 10:**
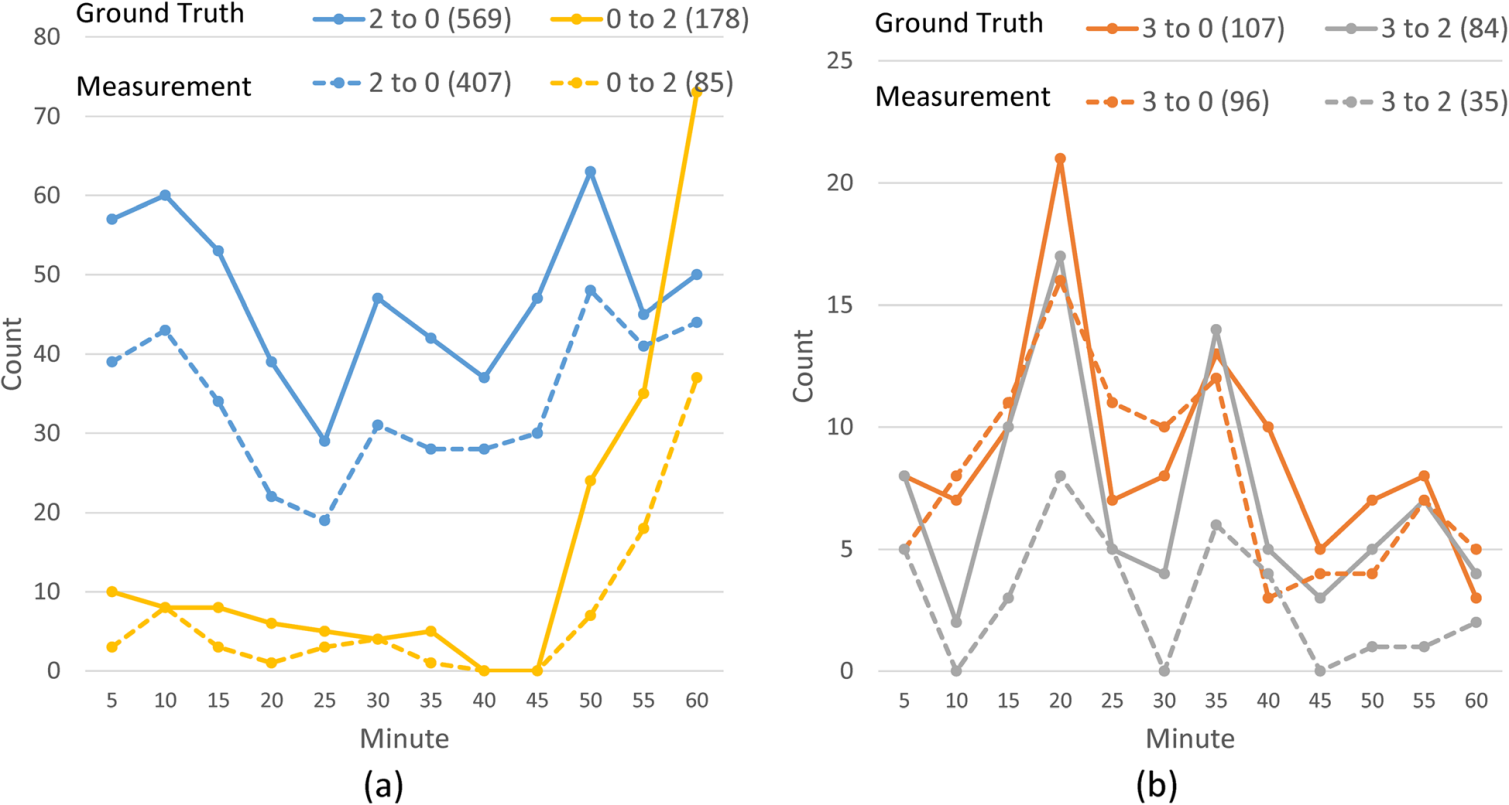
Intersection turning pattern after the TAMU vs. Florida game. Legend: origin to destination edge (total count). Solid lines represent the ground truth with 5 min interval, and dash lines indicate the measurement

Given the optimized matrices of samples 1 through 12, the change of one specific element over time, e.g., the number of vehicles moving from edge 2 to 0, is plotted in Fig. 10. Out of all 16 matrix elements, 12 of them (i.e., enter and leave combinations) have less than 15 counts after summing up all 12 samples, hence totaling 144 small data points. This brings difficulty of large error percentile variations and small number visualization. Therefore, when producing these plots, entries that have less than 15 total vehicle count over a 1-h time period are removed. The results show two main clusters: thru (2/0 and 0/2) and turn (3/0 and 3/2), shown with dash lines in Fig. 10a and b, respectively. Moreover, the removed edge combinations are consistent with the on-site traffic control. For example, edge 1 is blocked except for game-related vehicles (Texas A&M Transportation Institute, 2020) and no edge 1 in those two clusters. In these plots, the ground truth data are shown with solid lines for comparison. Using Eq. 7, the measurement and ground truth data are used to calculate the error for specific edge combinations. For example, the error percentage of edge 2 to 0 is 28%, and 52% for edge 0 to 2. Moreover, the traffic trend is successfully extracted. For example, traffic from edge 0 to 2 is observed to increase rapidly after minute 45. The same pattern is found in the ground truth data due to the removal of another traffic control (one lane was closed). Similarly, the trend is found to be consistent from edge 2 to 0, 3 to 0, and 3 to 2. On the other hand, Fig. 9b suggests a 29% error for the traffic from edge 3 to 0, and a 56% error for edge 3 to 2. The traffic plan allows only vehicles from Kimbrough Blvd. (approach edge 3) to turn to edge 0 or 2. The proposed framework observes more traffic turns to edge 0 than edge 2, which is consistent with the ground truth data. The number of vehicles and their turning pattern is important information to guide the traffic control in real-time (the number of vehicles approaching from Kimbrough Blvd. is known from pregame parking counts). This information can also be deployed as part of post-event reviews to adjust traffic control as well as game day parking arrangements.

## Conclusion

This paper proposed a computer vision based framework to automatically retrieve traffic information in large events. In particular, an object detection model (i.e., YOLOv5) and a tracking algorithm (i.e., Deep-SORT retrained on VeRi dataset) were used to track vehicles in video frames. The pixel tracks in the perspective camera view were consequently projected onto an orthogonal UTM map along with information including vehicle ID, location, and timestamp. This information was saved and further post-processed to provide accurate traffic volume counts. Videos recorded on a regular day from TAMU campus was manually labeled and used to investigate the influencing factors such as input size, light condition, camera angle, and pixel size. Experiments showed that the framework was capable of producing the traffic volume count with a 3.09% error and a 5.07 RSS (study Area 2, best out of 5 study areas) when benchmarked against human annotations. Based on the experiment analysis, it is also recommended to use an input size of 1280*1280, set separate thresholds for dark and bright frames, and set the study area so the detections have pixel size greater than 2343 and a camera angle below 22^°^. The framework outcome with these recommendations should achieve a similar performance as study Area 1, 2, and 3, which have an accuracy ranging from 93.38% to 96.91% and a framework speed of 2.09–3.6 FPS on a regular office computer. Besides, better GPU systems have the potential to process the monitoring video (15 FPS) at a real-time speed. Moreover, traffic volume data measured on University Drive after 4 home games (TAMU vs. Vanderbilt, Florida, Arkansas, and Louisiana State) in the 2020 football season was aggregated with a 5-min interval to allow in-depth analysis. The results showed consistency compared with the 2014 to 2020 season TTI reports about the game day traffic congestions.

Moreover, this work designed the intersection turning patterns and its optimization, which was not available before. The traffic turning matrix with counts for each ingress-egress edge pair revealed congestion development, especially left turns which generate delays. Considering the high performance of the traffic volume count previously, the edge total entering and leaving volumes are used as constraints to optimize the traffic turning matrix. In detail, 12 intersection turning pattern samples were created and annotated as the ground truth and the follow-up comparison suggested a 43% to 72% accuracy from the proposed framework. Moreover, the proposed intersection turning pattern revealed traffic change trends, consistent with the on-site traffic control. Last but not the least, the turning pattern experiment suggested that dark-colored vehicle crossing over dark-colored pavement introduces difficulty to vehicle tracking precision. Altogether, given the experiments and measurements, the proposed framework is an accurate and prompt method to extract traffic information from monitoring videos in major events. The research results can also provide benchmarks for future researchers to compare and improve.

The proposed framework is a more convenient, flexible, and affordable model than traditional traffic monitoring methods that rely on traffic sensors, GPS data, and in-situ human observation. Specifically, it is suggested by the experiment results that this framework works for various light conditions, i.e., suitable for long-term monitoring. The framework is generalizable to other cameras by manually selecting four reference points. Selecting the reference points is a convenient process that only requires one camera frame and an online UTM map. Moreover, the study areas can be changed flexibly according to traffic study needs such as different directions or combined lanes. In the urban environment, the framework is also applicable to other classes such as pedestrians, buses, and trucks. Lastly, this framework utilizes the existing traffic monitoring cameras, computers, and open source software (Python) which makes it affordable. Altogether, this framework can be applied in large events that draw significant vehicular traffic for multiple purposes. For example, real-time or near real-time traffic monitoring from this framework can support fast emergency response. Based on the historical data and mathematical modeling, the near future traffic can be forecasted. The recorded data can be used for agent-based traffic modeling hence support traffic planning and policy making. Different intersections can be monitored and compared to identify the congestion development, hence support the prevention and mitigation of such developments.

However, there are some limitations of this work which could be the future work directions. Firstly, this work examined vehicle counting and intersection turning, which are both counting-based data. The retrieved metadata (ID, position, and timestamp) can support more traffic statistics such as vehicle speed, lane change, and driving behavior (e.g., traffic rule compliance), which are also important indicators of road and operation safety. For example, it is possible to detection the traffic light in camera view and sense the vehicles running red lights. Secondly, it is observed that the framework underestimates in both the traffic volume count and intersection turning pattern experiments. To remedy this, more traffic count ground truth data could be created to compute the systematic bias, thus compensate the undercount. This ground truth data collection could benefit from the traditional traffic data collection techniques such as induction loops or radars. Another possibility is to use a multi-camera system to count the same/connected study areas and then use Bayesian inference to approximate the true count. The integration of multiple cameras from different locations in the city could deliver the bigger picture of the traffic flow, which otherwise is difficult to capture with a single camera. Such connected data will support systematical error correction and optimization as well as adaptive and data-driven parking arrangement. Lastly, even the 24-h-video showed the framework performance under various lighting conditions, there is still a lack of study of challenging weather such as snowing and raining, which might affect the visual appearance of the vehicles.

## Data Availability

Not applicable.
